# Sleep Quality and Sleep Health Before and After Hip or Knee Arthroplasty: A Prospective Cohort Study

**DOI:** 10.3390/jcm15103585

**Published:** 2026-05-07

**Authors:** Jordi Colomina Morales, Adriano D. S. Targa, Mario Henríquez-Beltrán, Esther Gracia-Lavedán, Iván Juez-Garcia, Jordi de Batlle

**Affiliations:** 1 Department of Orthopedic Surgery, University Hospital Santa Maria, Gestió de Serveis Sanitaris, 25198 Lleida, Spain; jcolomina@gss.cat; 2 Multidisciplinary Research Group in Musculoskeletal Pathology, Fragility and Pain Treatment, Institut de Recerca Biomèdica de Lleida—Fundació Dr. Pifarré, IRBLleida (ROR: 03mfyme49), 25198 Lleida, Spain; 3 Group of Translational Research in Respiratory Medicine, Institut de Recerca Biomèdica de Lleida—Fundació Dr. Pifarré, IRBLleida (ROR: 03mfyme49), 25198 Lleida, Spain; atarga@irblleida.cat (A.D.S.T.); eliashbm@hotmail.com (M.H.-B.); egracialavedan@gmail.com (E.G.-L.); ijuez@irblleida.cat (I.J.-G.); 4 Centro de Investigación Biomédica en Red de Enfermedades Respiratorias (CIBERES), 28029 Madrid, Spain; 5 Magíster en Ciencias de la Motricidad Humana, Universidad Adventista de Chile, Chillán 3780000, Chile

**Keywords:** sleep, sleep quality, osteoarthritis, knee arthroplasty, hip arthroplasty

## Abstract

**Background/Objectives:** Sleep quality is a critical determinant of recovery after total joint arthroplasty (TJA), yet multidimensional trajectories of sleep health remain poorly defined. This study aimed to describe the trajectory of sleep health and sleep quality before and after hip or knee arthroplasty, and to examine the influence of sex and surgical site. **Methods:** A single-centre, prospective cohort study was conducted including 316 patients scheduled for primary hip or knee arthroplasty. Sleep was assessed two months before surgery and at one and six months postoperatively, using the Pittsburgh Sleep Quality Index (PSQI) and the RU-SATED scale. Statistical analyses used linear mixed-effects models with random intercepts for participants. Effect sizes (Cohen’s d) were derived from the residual variance of the mixed models. **Results:** At baseline, 40% of patients had poor sleep quality. Estimated marginal mean PSQI scores were 5.30 (95% CI: 4.87 to 5.73) preoperatively, 5.71 (5.27 to 6.15) at one month, and 4.19 (3.73 to 4.65) at six months, representing a reduction of 1.11 points (95% CI: 0.69 to 1.54; Cohen’s d = 0.49, 95% CI: 0.30 to 0.68; *p* < 0.001) from baseline to six months. The proportion of patients with poor sleep quality (PSQI > 5) fell from 40% to 28%, a 12 percentage-point absolute reduction reflecting individual-level transitions across the validated clinical threshold. Exploratory component analyses showed improvements in subjective sleep quality, sleep duration, sleep efficiency, and sleep disturbance. RU-SATED scores increased from 9.72 (95% CI: 9.46 to 9.98) to 10.45 (10.19 to 10.72) at six months, an improvement of 0.73 points (95% CI: 0.47 to 1.00; Cohen’s d = 0.44, 95% CI: 0.27 to 0.60; *p* < 0.001). Women undergoing knee arthroplasty had significantly worse sleep scores than men at baseline and one month postoperatively, though both sexes followed parallel recovery trajectories (time-by-sex interaction *p* > 0.30), with the absolute sex difference narrowing by six months. In hip arthroplasty, no significant sex differences or time-by-sex interactions were observed after adjusting for the age imbalance between sexes. **Conclusions:** Arthroplasty was associated with significant improvements in multidimensional sleep health by six months, though the first postoperative month represents a period of stagnation or slight decline. Women undergoing knee arthroplasty consistently reported worse sleep than men, although the recovery trajectory was parallel between sexes. These findings highlight the potential value of integrating sleep assessment into perioperative care, particularly for women scheduled for knee arthroplasty, though whether targeted sleep interventions improve clinical outcomes remains to be established through prospective intervention studies.

## 1. Introduction

Osteoarthritis affects approximately 7.6% of the global population, with approximately 61% of cases occurring in women [1,2]. At its end stage, total hip arthroplasty (THA) and knee arthroplasty (KA), either total (TKA) or unicompartmental (UKA), are among the most effective surgical treatments. The number of THA have risen by 30% and KA by 40% across OECD countries, between 2007 and 2017 [3]. While Enhanced Recovery After Surgery (ERAS) protocols have improved outcomes by reducing complications and hospital stays [4,5], postoperative pain and functional recovery remain major concerns [6].

Sleep plays a crucial role in general well-being and postoperative recovery and is intertwined with postoperative outcomes in patients undergoing arthroplasty [7,8]. Poor sleep is associated with heightened pain sensitivity and impaired healing [9,10]. Preoperative sleep disturbances have been linked to worse pain control at six months after THA [11], and sleep disruption has been shown to reduce the efficacy of opioid analgesia [12]. Moreover, good preoperative sleep quality has been associated with shorter hospital stays [13] and better functional outcomes [14] in TKA and THA patients within ERAS programmes.

Current research on sleep in the context of joint arthroplasty remains limited. First, most studies assess sleep only at hospital admission, which may not reflect habitual sleep patterns in the period leading up to the surgery procedure. Second, most studies include limited assessments of sleep quality, relying on excerpts from the Pittsburgh Sleep Quality Index (PSQI) [15], and just a few [14,16] have used multidimensional scales like the RU-SATED scale [17], which defines optimal sleep as more than the mere absence of disease. Third, longitudinal data on sleep health trajectories are scarce (see for example the works from Manning [18] and Van Meirhaeghe [19]). Fourth, few studies extend follow-up to six months postoperatively while also capturing a true preoperative baseline. Finally, small sample sizes have limited the ability to explore the combined effects of sex and surgical site on both sleep health and recovery [10,18].

Sex and surgical site are potential sources of variation in sleep recovery, given documented sex differences in pain sensitivity and osteoarthritis prevalence, and differences in postoperative pain profiles between hip and knee procedures. The present study aimed to describe the full trajectory of sleep quality and health in patients undergoing primary hip or knee arthroplasty from 2 months preoperative to 6 months postoperative and to explore how sleep outcomes varied according to sex and surgical site in descriptive subgroup analyses.

## 2. Materials and Methods

A single-centre, prospective cohort study involving patients undergoing primary THA and primary knee arthroplasty, encompassing both TKA and UKA, was conducted from February 2023 to March 2024 at the University Hospital of Santa Maria in Lleida, Spain. Patients were recruited consecutively during their preoperative anaesthetic visit, approximately two months before their scheduled surgery. Inclusion criteria were patients aged 18 years or older, scheduled for primary THA, TKA, or UKA. All procedures were unilateral; bilateral simultaneous procedures were not performed at this centre during the study period. Only primary (non-revision) arthroplasties were included. Exclusion criteria included fracture-related procedures, a psychiatric or cognitive condition precluding informed consent or independent questionnaire completion, inability to complete questionnaires, a life expectancy of less than one year, or declining to participate. All surgical procedures were performed by nine experienced surgeons. Patients received care based on the centre’s ERAS protocol, which included preoperative education, preemptive analgesia, spinal and local intraarticular anaesthesia, no tourniquet, no drains, mobilisation in the first 24 h after surgery, and a multimodal opioid sparing analgesia regime [4]. All surgeons followed the same standardised ERAS protocol. Patients with diagnosed sleep disorders were not explicitly excluded from the study; their sleep status at baseline is reflected in the PSQI and RU-SATED scores.

### 2.1. Patient Characterisation and Clinical Data

Data were collected at four distinct time points: at the preoperative anaesthetic visit, approximately two months before surgery (M_−2_), during surgery and hospitalisation (M_0_), after hospital discharge (M_1_), and six months after hospital discharge (M_6_). Preoperative and postoperative data were collected by trained nurses in outpatient visits. Surgical data were extracted from electronic clinical records. At each time point (M_-2_, M_1_, and M_6_), patients completed a series of questionnaires to provide a comprehensive profile. Postoperative assessments were conducted during scheduled clinical follow-up visits that form part of the standard ERAS care pathway at our centre, which contributed to retention rate. The three assessment time points were selected to coincide with the standard clinical follow-up visits in our centre’s ERAS care pathway, enabling data collection to be embedded within routine care with minimal additional burden on participants. The data collected included socio-demographic, anthropometric, and lifestyle variables, as well as information on their comorbidities. Diagnosis, classification and surgery data included the American Society of Anesthesiologists (ASA) physical status classification system for each diagnosis and any relevant surgical-related data.

### 2.2. Sleep Quality and Sleep Health Assessments

Both sleep quality and sleep health were quantified using two validated, patient-reported outcome measures, the PSQI [15] and the RU-SATED scale [17], respectively, to capture complementary aspects of participants’ sleep experiences and behaviours. Details of the questionnaire items are provided in the Supplementary Materials. Sleep quality is defined as an individual’s self-satisfaction with all aspects of the sleep experience. It includes attributes such as sleep efficiency, sleep latency, sleep duration, and wake after sleep onset [20]. In contrast, sleep health is a multidimensional concept that refers to a sleep–wakefulness pattern that adapts to individual, social, and environmental demands to promote physical and mental well-being [21].

The PSQI assessed subjective sleep quality across seven dimensions: subjective sleep quality, sleep latency, sleep duration, habitual sleep efficiency, sleep disturbances, use of sleep medication, and daytime dysfunction. Each dimension was scored on a scale from 0 to 3, yielding a global score ranging from 0 to 21. In accordance with the original validation [15], a global score greater than 5 was considered indicative of poor sleep quality. Given the one-month recall period of the PSQI, the M_1_ assessment captured sleep during the post-discharge home recovery phase rather than the hospitalisation itself.

The RU-SATED scale [17] was used as a multidimensional tool to assess sleep health across six core dimensions: Regularity (consistency of sleep schedule), Satisfaction (subjective sleep quality), Alertness (daytime functioning), Timing (synchronisation with the optimal time to sleep), Efficiency (proportion of time spent asleep while in bed), and Duration (total sleep time). The overall RU-SATED score was calculated using the original validated scoring, with each of the six dimensions scored from 0 to 2, yielding a total score ranging from 0 to 12, where higher scores reflect better sleep health [17]. For dimension-level analyses, each item was scored using all five available Likert response options (0 = never, 1 = rarely, 2 = sometimes, 3 = often, 4 = always), yielding a per-dimension range of 0 to 4. This extended scoring format was used to increase sensitivity in detecting within-dimension change but has not been independently validated for this population. The dimension-level results should therefore be interpreted as exploratory only, and are not currently suitable as a basis for deriving clinical cutoffs or interpretive thresholds for individual sleep health dimensions.

### 2.3. Statistical Analysis

The primary outcomes were the PSQI global score and the RU-SATED global score. Individual PSQI component and RU-SATED dimension analyses were exploratory. Subgroup analyses by sex and surgical site were exploratory and not pre-specified for interaction testing.

Continuous variables were summarised using means (standard deviations (SDs)) and categorical variables as absolute and relative frequencies. For each primary outcome, a linear mixed-effects model was fitted with time (three-level factor: M_−2_, M_1_, M_6_) as a fixed effect and a random intercept for participant, including all available observations under the assumption of missing at random. Estimated marginal means with 95% confidence intervals, Bonferroni-adjusted pairwise comparisons, and effect sizes (Cohen’s d, derived from the residual standard deviation) are reported. For PSQI components, non-parametric sensitivity analyses (Wilcoxon signed-rank tests) were performed. Subgroup models included time, sex, and their interaction as fixed effects. For the hip subgroup, age was included as a covariate in models where it was a significant predictor. Sensitivity analyses excluded UKA cases to assess robustness of knee group results.

No formal a priori sample size calculation was performed and the study was not prospectively registered in a clinical trial registry; the study recruited all consecutive eligible patients over a full calendar year to ensure seasonal representativeness. For descriptive and component-level analyses, complete-case analysis was used; missingness for baseline variables did not exceed 3%, with the exception of baseline PSQI (available for 259 of 316 patients).

Given that no validated MCID has been established for the RU-SATED, score changes are discussed in terms of effect size. For the PSQI, the estimated change is interpreted in relation to the MCID of 3 points proposed by Longo et al. [22], alongside the reduction in the proportion of patients classified as poor sleepers (PSQI > 5) as a complementary indicator of clinical relevance.

R statistical software, version 4.4, was used for all analyses.

### 2.4. Ethics

All participants provided written informed consent prior to enrolment. The study was approved by the University Hospital Arnau de Vilanova Ethics Committee, with approval number CEIC-2716. The study was conducted in full compliance with the General Data Protection Regulation, the Declaration of Helsinki, and other applicable laws and regulations.

## 3. Results

Of the 347 eligible patients, 27 declined participation, and four did not attend at least one postoperative follow-up visit; thus, 316 were included in the analysis. RU-SATED data were available for 316 patients at M_−2_, 306 at M_1_, and 297 at M_6_ (287 [90.8%] with data at all three visits). PSQI data were available for 259 patients at M_−2_, 236 at M_1_, and 204 at M_6_ (181 [69.9%] with data at all three visits). The study population comprised 95 THA cases (30.1%) and 221 primary knee arthroplasties (69.9%), including 22 UKA and 199 TKA (48 of them being robotic-assisted). Among knee arthroplasty patients, 141 (63.8%) were women and 80 (36.2%) men, with a mean age of 71.0 years (SD 7.92) and mean BMI of 31.5 (SD 5.37). For hip arthroplasty, 43 (45.3%) were women and 52 (54.7%) men, with a mean age of 65.0 years (SD 12.4) and mean BMI of 29.1 (SD 5.06). Detailed patient characteristics are presented in Table 1.

### 3.1. Overall Sleep Health and Sleep Quality Trajectories

**Figure 1:**
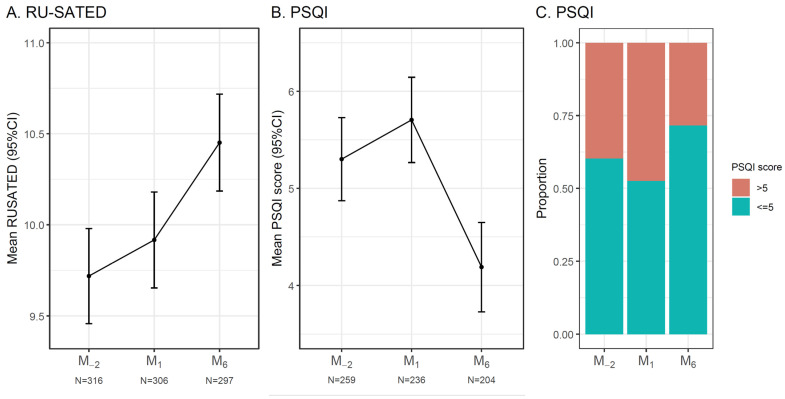
RU-SATED sleep health scale and the Pittsburgh Sleep Quality Index (PSQI) scores from two months before surgery (M_−2_) to one and six months after surgery (M_1_ and M_6_). Values represent estimated marginal means (95% CI) derived from a linear mixed-effects model with a random intercept for participant. Pairwise comparisons revealed significant differences between M_6_ and both previous visits (M_−2_ and M_1_; *p* < 0.001) for both questionnaires, while no significant differences were observed between M_−2_ and M_1_. (**A**) Mean RU-SATED score; (**B**) mean PSQI score; (**C**) proportion of participants reporting good sleep quality (PSQI score ≤ 5).

The RU-SATED showed estimated marginal mean scores of 9.72 (95% CI: 9.46 to 9.98) at M_-2_, 9.92 (9.65 to 10.18) at M_1_, and 10.45 (10.19 to 10.72) at M_6_ (overall time effect F = 15.19, *p* < 0.001). The change from M_−2_ to M_1_ was not statistically significant (difference 0.20, *p* = 0.43), whereas the improvement from M_-2_ to M_6_ was significant (difference 0.73, 95% CI: 0.47 to 1.00, Cohen’s d = 0.44, 95% CI: 0.27 to 0.60, *p* < 0.001), as was the change from M_1_ to M_6_ (difference 0.54, *p* < 0.001). Regarding the PSQI, estimated marginal mean scores were 5.30 (95% CI: 4.87 to 5.73) at M_−2_, 5.71 (5.27 to 6.15) at M_1_, and 4.19 (3.73 to 4.65) at M_6_ (overall time effect F = 24.09, *p* < 0.001). The change from M_−2_ to M_1_ did not reach significance (difference 0.40, *p* = 0.15), whereas the improvement from M_−2_ to M_6_ was significant (difference 1.11, 95% CI: 0.69 to 1.54, Cohen’s d = 0.49, 95% CI: 0.30 to 0.68, *p* < 0.001), as was the change from M_1_ to M_6_ (difference 1.52, *p* < 0.001). When considering the established PSQI threshold (scores > 5 indicating poor sleep quality), 40% of patients had poor preoperative sleep quality, decreasing to 28% at six months. By surgical site, the baseline prevalence of poor sleep quality was 38.3% (95% CI: 31.2% to 45.9%) for knee arthroplasty and 43.0% (95% CI: 31.9% to 54.7%) for hip arthroplasty. Women had higher rates of poor baseline sleep quality than men at both sites (knee: 45.7% vs. 25.0%; hip: 52.8% vs. 34.9%) (Figure 1).

### 3.2. Sleep Health and Sleep Quality Dimensions

**Figure 2:**
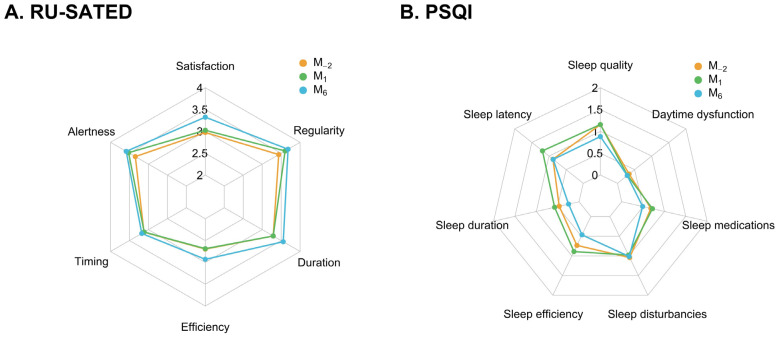
Evolution of the RU-SATED sleep health scale dimensions and the Pittsburgh Sleep Quality Index (PSQI) dimensions from two months before surgery (M_-2_) to one and six months after surgery (M_1_ and M_6_): (**A**) mean score in the RU-SATED dimensions across time; (**B**) mean score in the PSQI dimensions across time.

The following component- and dimension-level results are exploratory and should be interpreted with caution given the number of comparisons involved. The RU-SATED scale showed significant improvements over time in four of six dimensions when comparing baseline (M_−2_) to six months after surgery (M_6_). Satisfaction improved from 2.97 (1.22) to 3.33 (0.98), *p* < 0.001; Alertness from 3.34 (1.16) to 3.59 (0.87), *p* < 0.001; Duration from 3.29 (1.28) to 3.56 (0.96), *p* < 0.001; and Regularity from 3.44 (1.00) to 3.68 (0.74), *p*< 0.001. Neither Timing nor Efficiency improved significantly over the study period. Timing showed negligible change (from 3.12 [1.13] to 3.18 [1.08], *p* = 0.5). Although Efficiency increased from 2.70 (1.44) to 2.93 (1.35), this change did not meet the Bonferroni-corrected significance threshold (*p* = 0.02 vs. threshold *p* < 0.017). The PSQI component analysis revealed improvements from M_−2_ to M_6_ in subjective sleep quality, from 1.15 (0.71) to 0.88 (0.70), *p* < 0.001; in sleep duration, from 0.46 (0.78) to 0.25 (0.58), *p* = 0.009; and in sleep efficiency, from 0.73 (1.07) to 0.47 (0.81), *p* < 0.001. Regarding sleep disturbance, the change from 1.04 (0.44) to 1.01 (0.29), although statistically significant (*p* = 0.013), was clinically negligible (0.03 points on a 0–3 scale) and likely reflected the large sample size rather than a meaningful shift. Medication use showed a reduction from 0.68 (1.23) to 0.49 (1.10) (parametric *p* = 0.016; Wilcoxon *p* = 0.020); this did not consistently reach the Bonferroni-corrected threshold across both approaches. Other PSQI dimensions, including latency and daytime dysfunction, did not show significant changes. The Spider plots showing changes in the dimensions’ scores across time are shown in Figure 2. Sensitivity analyses using Wilcoxon signed-rank tests for all PSQI components yielded *p*-values consistent with the parametric results, with no change in the pattern of significance for any component (Supplementary Materials).

### 3.3. Subgroup Analyses by Sex and Surgical Site

When stratified by sex and surgical site, mixed-effects models with time-by-sex interaction terms revealed parallel recovery trajectories for both sexes (all interactions: *p* > 0.30).

**Figure 3:**
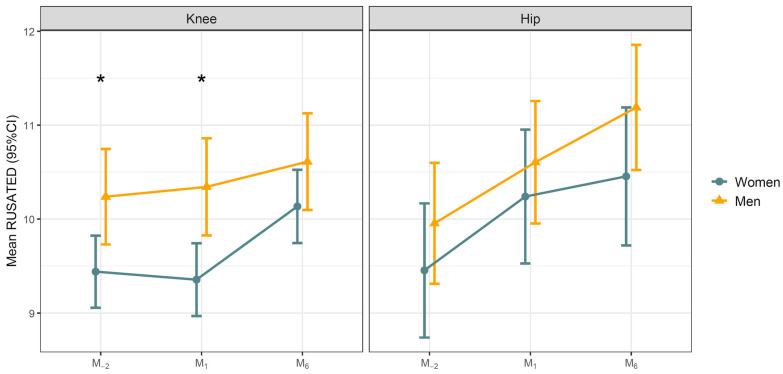
RU-SATED sleep health scores by surgical site and sex. Values represent estimated marginal means (95% CI) derived from a linear mixed-effects model including visit, sex, and their interaction, with a random intercept for participant. The hip arthroplasty model was additionally adjusted for age. Pairwise comparisons between sexes within each visit were Bonferroni-adjusted. Statistically significant differences are indicated by an asterisk (*).

**Figure 4:**
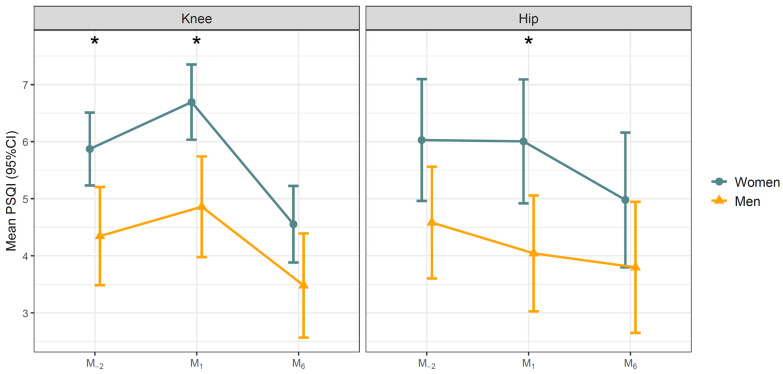
Pittsburgh Sleep Quality Index (PSQI) scores by surgical site and sex. Values represent estimated marginal means (95% CI) derived from a linear mixed-effects model including visit, sex, and their interaction, with a random intercept for participant. Pairwise comparisons between sexes within each visit were Bonferroni-adjusted. Statistically significant differences are indicated by an asterisk (*).

For knee arthroplasty, the time-by-sex interaction was not significant for either the RU-SATED (*p* = 0.32) or the PSQI (*p* = 0.40), indicating that both sexes followed comparable patterns of change over time. However, pairwise between-sex comparisons within each visit confirmed that women had significantly worse scores than men at M_−2_ and M_1_. For the RU-SATED, the estimated sex difference was 0.80 points at M_-2_ (*p* = 0.014) and 0.99 points at M_1_ (*p* = 0.003), narrowing to 0.48 points at M_6_ (*p* = 0.15). For the PSQI, women scored 1.53 points higher (worse) than men at M_−2_ (*p* = 0.005) and 1.83 points higher at M_1_ (*p* = 0.001), with the difference narrowing to 1.07 points at M_6_ (*p* = 0.063). Estimated marginal means by sex and visit are shown in Figure 3 and Figure 4.

For hip arthroplasty, the RU-SATED model was adjusted for age given the significant age imbalance between sexes. Age was a significant predictor (beta = 0.04 per year, *p* = 0.016). The time-by-sex interaction was not significant (*p* = 0.77), and no significant between-sex differences were observed at any visit after age adjustment. For the PSQI, the overall effect of time did not reach statistical significance in the hip group (*p* = 0.067), and the time-by-sex interaction was non-significant (*p* = 0.59). The limited sample sizes in the hip subgroups (n = 43 women, n = 52 men) preclude definitive conclusions about the absence of sex effects.

Sensitivity analyses excluding the 22 UKA cases and restricting the knee group to TKA procedures (n = 199) yielded virtually identical results for all models (Supplementary Materials).

## 4. Discussion

This prospective cohort study of 316 patients undergoing primary hip or knee arthroplasty describes how both sleep quality and multidimensional sleep health follow a consistent perioperative trajectory: a modest, non-significant decline in the first postoperative month, followed by significant improvement by six months. Preoperatively, 40% of patients presented with poor sleep quality as measured by the PSQI; this proportion fell to 28% at six months, representing a clinically meaningful reduction in the burden of sleep disturbance in this population. RU-SATED scores improved from 9.7 (2.5) at baseline to 10.5 (2.1) at six months (*p* < 0.001). Improvements were observed across both instruments, predominantly in the knee arthroplasty cohort. Notably, the overall PSQI improvement was driven by the knee group; in the hip group, the effect of time on PSQI scores did not reach statistical significance (*p* = 0.067), whereas the RU-SATED showed significant improvement in both groups. Women undergoing knee arthroplasty presented with the worst baseline sleep and consistently scored worse than men, though both sexes followed parallel recovery trajectories. Sex differences in the hip arthroplasty cohort were minimal, with both sexes showing comparable and significant improvement by six months. To our knowledge, this is among the first studies to apply the RU-SATED scale in an arthroplasty population, enabling assessment of sleep as a multidimensional health behaviour rather than as a symptom alone, and the findings suggest that joint replacement has a broad and positive impact on sleep that extends beyond the reduction in pain-related nocturnal disturbance. All findings reported here concern subjective, patient-reported dimensions of sleep quality and sleep health; objective sleep architecture, including sleep stages, arousals, and respiratory events, was not assessed, and the conclusions of this study should be read within that scope.

Results at baseline showed that 40% of patients exhibited poor sleep quality according to the PSQI. Sleep quality slightly worsened at one month after surgery, but improved significantly by six months, with 28% of patients maintaining poor sleep quality. The estimated PSQI improvement of 1.11 points (95% CI: 0.69 to 1.54) from baseline to six months falls below the MCID of 3 points proposed by Longo et al. [22]; however, that threshold derives from a single study in a different surgical population, and no consensus MCID for the PSQI has been established in the arthroplasty setting. We consider the 12 percentage-point absolute reduction in the proportion of patients classified as poor sleepers (from 40% to 28%) to be a more clinically meaningful indicator than the modest change in mean PSQI score, since it reflects individual-level transitions across the validated clinical threshold (PSQI > 5) rather than a small shift in a continuous mean. Approximately 30% of patients classified as poor sleepers preoperatively had crossed into the good-sleeper category by six months, an effect that is likely to be more interpretable to clinicians than a sub-MCID change in mean global score. Regarding sleep health, the RU-SATED increased significantly from a baseline estimated marginal mean of 9.72 to 10.45 at six months, though given the residual variability, a change of 0.73 points corresponds to a Cohen’s d of 0.44 (95% CI: 0.27 to 0.60). Under conventional thresholds, this falls in the small-to-medium range; however, recently proposed field-specific cutoffs for sleep interventions (small = 0.18, medium = 0.33, large = 0.56) [23] suggest that both the RU-SATED (d = 0.44) and PSQI (d = 0.49) effects fall in the medium-to-large range relative to what is typically observed in sleep research. These empirically derived cutoffs were obtained from randomised controlled trials of sleep-improving interventions on mental health outcomes rather than observational surgical cohorts, and should therefore be considered an approximate rather than definitive benchmark. To our knowledge no validated MCID for the RU-SATED exists and the clinical significance of this magnitude of change remains to be established in future work. Improvements were observed predominantly in patients undergoing knee arthroplasty, with women consistently reporting worse sleep scores than men, though both sexes followed parallel recovery trajectories over time.

Our findings align with and extend the current body of literature on this subject [10]. The observed overall prevalence of poor sleep quality at baseline (40%) is consistent with previous reports [19]. By surgical site, the baseline prevalence in our cohort was 38.3% (95% CI: 31.2% to 45.9%) for knee arthroplasty and 43.0% (95% CI: 31.9% to 54.7%) for hip arthroplasty. Van Meirhaeghe et al. [19] reported that 30% of knee arthroplasty patients and 39% of hip arthroplasty patients had poor preoperative sleep quality, figures broadly comparable to ours, although our hip prevalence was somewhat higher. Others [16] reported that up to 60% of patients scheduled for total hip or knee arthroplasty met the criteria for sleep disturbance before surgery. Prior studies have documented transient postoperative sleep disruption, particularly in the early recovery phase [18,24]. Our study adds longitudinal depth by demonstrating significant improvements in both sleep quality and sleep health from the first to the sixth month post-surgery.

Studies have relied primarily on the PSQI [11,13,14,18,19,24]; our study adds a novel contribution by incorporating the RU-SATED scale to assess multidimensional sleep health. Sleep quality, as captured by the PSQI, is assessed through self-reported sleep disturbances and difficulties commonly linked to health conditions, such as pain, discomfort, and hospital-related environmental disruptions. In contrast, sleep health, as measured by RU-SATED, reflects a multidimensional pattern of sleep behaviours and quality that influences systemic outcomes including emotional regulation, cognitive functioning, and overall well-being [17,25]. The joint use enabled a comprehensive understanding of sleep as both a biological process and a health behaviour.

Koken and Guclu [26] previously examined PSQI scores before and at six weeks and six months after TKA in 80 patients, finding a non-significant worsening at six weeks followed by a statistically significant improvement in total PSQI score at six months (7.1 to 5.2, *p* < 0.001). Significant gains were restricted to the daytime dysfunction and overall sleep quality subdimensions, while sleep disturbances, sleep latency, and efficiency showed no significant change, and no association was found between sex and PSQI scores at any time point. Our findings in a larger, prospective cohort that includes both TKA and THA patients extend this work. The overall trajectory we observed is consistent, providing prospective confirmation of a pattern first identified retrospectively. Our exploratory PSQI component analysis identified improvements in subjective sleep quality, sleep efficiency, and sleep duration. However, component-level findings such as sleep disturbance (a change of 0.03 points on a 0–3 scale) should be interpreted with caution, as statistical significance in large samples does not imply clinical relevance. Beyond replicating the PSQI trajectory, our study documents improvements across four of six RU-SATED domains, reflecting positive changes in sleep as a broader health behaviour rather than as a symptom-driven complaint alone, a dimension that a purely PSQI-based retrospective study could not capture.

Exploratory subgroup analyses revealed that women undergoing knee arthroplasty had significantly worse sleep scores than men at baseline and at one month postoperatively for both instruments. However, formal time-by-sex interaction tests were non-significant for both the RU-SATED (*p* = 0.32) and the PSQI (*p* = 0.40) in the knee group, indicating that both sexes followed parallel recovery trajectories over time. The absolute sex difference narrowed descriptively by six months, at which point it was no longer statistically significant. These findings suggest a consistent sex difference in the absolute burden of sleep disturbance rather than a differential response to surgery. In the hip arthroplasty group, no significant sex differences or time-by-sex interactions were observed for either instrument after adjustment for the significant age imbalance between sexes (mean 69.6 years in women vs. 61.3 in men, *p* = 0.001). However, the limited sample sizes (n = 43 women, n = 52 men) preclude definitive conclusions. The female predominance in the knee group (63.8%) means that overall knee results are weighted towards the female experience, and this should be considered when interpreting the knee-specific findings. The higher prevalence of poor baseline sleep quality in women (knee: 45.7% vs. 25.0%; hip: 52.8% vs. 34.9%) is consistent with Van Meirhaeghe et al. [19], who found that women reported higher rates of poor preoperative sleep quality than men. Our findings extend this by showing that the between-sex difference persists into the early postoperative period but narrows by six months, though whether this convergence reflects differential recovery rates or regression to the mean cannot be determined from these data. Osteoarthritis of the knee is more prevalent among women, constituting 63.8% of the knee arthroplasty cases in our cohort. Postoperative pain following knee arthroplasty tends to be more severe and prolonged than after hip arthroplasty, which may contribute to greater sleep disturbance in this group [27,28]. Nandi et al. [29] reported that women had greater baseline pain-related physical dysfunction and higher acute postoperative pain scores in the first two weeks following TKA than men, with sex differences in reported pain resolving by six weeks postoperatively, a temporal pattern that closely parallels the narrowing of sleep score differences between sexes observed in our cohort. Additional hormonal and psychosocial factors may further exacerbate the pain–sleep cycle in female patients [6,30], though these were beyond the scope of the present study.

A distinguishing feature of this study is the employment of two rigorously validated instruments to assess both subjective sleep quality and multidimensional sleep health. The inclusion of RU-SATED, which is rarely used in arthroplasty research, provided a broader and more integrative perspective on sleep both as a biological process and a health behaviour. The prospective design, large sample size, and repeated assessments before and after surgery allowed for a robust evaluation of sleep trajectories over time. Additionally, stratified analyses by sex and surgical site offered valuable insights into differential recovery patterns. Nonetheless, some limitations should be acknowledged. First, the study relied exclusively on patient-reported outcomes, without objective sleep measures such as actigraphy or polysomnography. Subjective assessments are inherently susceptible to recall bias and individual interpretation. Second, the single-centre design within a specific ERAS pathway limits the generalisability of findings to non-ERAS settings. Multimodal opioid-sparing analgesia and early mobilisation, two core components of the protocol applied at our centre, are specifically designed to minimise the perioperative factors most disruptive to sleep, namely uncontrolled pain and prolonged immobility. The favourable mid-term sleep trajectories observed here may therefore reflect, at least in part, the protective effect of the ERAS pathway itself, and the magnitude of postoperative recovery should not be assumed to extrapolate to settings using traditional perioperative care. Third, although pain scores and analgesic use data were collected as part of the study protocol, a comprehensive analysis of these outcomes is being prepared as a separate manuscript. Because postoperative pain and opioid exposure both directly disrupt sleep continuity and architecture, these unmeasured factors are plausible confounders of the perioperative sleep trajectories reported here, and their role as confounders or mediators will be examined formally in that separate analysis. Fourth, the extended 0–4 scoring format used for RU-SATED dimension-level analyses has not been independently validated for this population; these dimension-level results should be considered exploratory and are not yet suitable for deriving clinical cutoffs or interpretive thresholds at the dimension level. Fifth, the PSQI improvement of 1.11 points falls below the 3-point MCID proposed by Longo et al. [22], though the 12 percentage-point reduction in patients classified as poor sleepers may be a more clinically meaningful indicator. Sixth, the significant age imbalance between sexes in the hip group (*p* = 0.001) was addressed statistically by including age as a covariate, but residual confounding cannot be excluded. Seventh, no formal a priori power calculation was performed; the sample was defined by the recruitment period rather than by a statistical target, and subgroup analyses, particularly in the hip arthroplasty group (n = 95), may be underpowered to detect small between-group differences. Eighth, this study was not prospectively registered in a clinical trial registry; the study protocol was approved by the institutional ethics committee prior to the start of data collection, providing independent oversight. Ninth, pre-existing sleep disorder diagnoses were not systematically recorded as a covariate, precluding examination of their influence on perioperative sleep trajectories; obstructive sleep apnoea, recorded as a comorbidity, was present in a small proportion of patients (5.1% overall) and was not adjusted for. Finally, follow-up was limited to six months, and longer-term trajectories remain to be characterised.

Our findings have clinical implications for the perioperative management of patients undergoing hip and knee arthroplasty. Sleep disturbances are very common in this population and negatively impact pain perception, emotional regulation, and functional recovery [11,18]. Our findings suggest that routine preoperative sleep assessment could identify patients with poor sleep quality, who constituted 40% of our cohort, especially women scheduled for knee arthroplasty. However, whether directly targeting sleep disturbance through pharmacological or behavioural interventions improves postoperative outcomes remains to be established through prospective intervention studies. Arthroplasty was associated with significant mid-term improvements in both sleep quality and sleep health. Exploratory component analyses suggested that PSQI improvements were most notable in subjective sleep quality and efficiency. RU-SATED scores increased across nearly all domains, highlighting the broader impact of surgery on sleep-related behaviours and physiology.

## 5. Conclusions

This study found that approximately 40% of patients undergoing primary hip or knee arthroplasty present with poor sleep quality before surgery, a proportion that falls to 28% by six months postoperatively. This 12 percentage-point reduction at the cohort level, corresponding to approximately 30% of baseline poor sleepers crossing into the good-sleeper category by six months, likely represents the most clinically meaningful signal in our data, more so than the modest change in mean PSQI score. Both sleep quality and multidimensional sleep health follow a consistent trajectory: a transient decline in the first postoperative month followed by significant improvement by six months. Women undergoing knee arthroplasty consistently reported worse sleep scores than men, although both sexes followed parallel recovery trajectories. Given the well-established negative impact of sleep disturbances on pain perception, emotional regulation, and functional recovery [11,18], our findings suggest an opportunity to integrate routine preoperative sleep assessment into perioperative care pathways, particularly for women scheduled for knee arthroplasty. Whether directly targeting sleep disturbance improves clinical outcomes in this population remains to be established through prospective intervention studies. Future research should also explore how sleep metrics relate to key orthopaedic outcomes including pain control, mobility, and rehabilitation progress, accounting for potential confounders such as pharmacological treatments, comorbidities, and surgical technique.

## Figures and Tables

**Table 1:** Baseline characteristics of patients by surgical site (hip vs knee) and sex.

	KNEE				HIP			
	All	Women	Men		All	Women	Men	
	N = 221	N = 141	N = 80	*p*-value	N = 95	N = 43	N = 52	*p*-value
Age, years	71.0 (7.92)	71.7 (6.99)	69.8 (9.27)	0.113	65.0 (12.4)	69.6 (11.4)	61.3 (12.0)	0.001
BMI, kg/m^2^	31.5 (5.37)	31.9 (5.77)	31.0 (4.61)	0.193	29.1 (5.06)	28.3 (4.78)	29.7 (5.23)	0.165
Smoking status				0.024				0.563
Former or never smoker	202 (92.2%)	133 (95.7%)	69 (86.2%)		83 (87.4%)	39 (90.7%)	44 (84.6%)	
Current smoker	17 (7.76%)	6 (4.32%)	11 (13.8%)		12 (12.6%)	4 (9.30%)	8 (15.4%)	
ASA classification				0.269				0.214
I	22 (10.2%)	17 (12.4%)	5 (6.33%)		13 (14.3%)	3 (7.32%)	10 (20.0%)	
II	145 (67.1%)	89 (65.0%)	56 (70.9%)		63 (69.2%)	32 (78.0%)	31 (62.0%)	
III	48 (22.2%)	31 (22.6%)	17 (21.5%)		14 (15.4%)	6 (14.6%)	8 (16.0%)	
IV	1 (0.46%)	0 (0.00%)	1 (1.27%)		1 (1.10%)	0 (0.00%)	1 (2.00%)	
Surgical procedure				0.051				
Total hip arthroplasty					95 (100%)	43 (100%)	52 (100%)	
Total knee arthroplasty	151 (68.3%)	105 (74.5%)	46 (57.5%)					
Unicompartmental knee arthroplasty	22 (9.95%)	12 (8.51%)	10 (12.5%)					
Robotic total knee arthroplasty	48 (21.7%)	24 (17.0%)	24 (30.0%)					
Surgical diagnosis				0.288				0.962
Primary osteoarthritis	180 (93.3%)	115 (92.7%)	65 (94.2%)		63 (74.1%)	29 (72.5%)	34 (75.6%)	
Avascular necrosis	5 (2.59%)	5 (4.03%)	0 (0.00%)		15 (17.6%)	8 (20.0%)	7 (15.6%)	
Post-traumatic osteoarthritis	5 (2.59%)	3 (2.42%)	2 (2.90%)		1 (1.18%)	0 (0.00%)	1 (2.22%)	
Rheumatic disease	2 (1.04%)	1 (0.81%)	1 (1.45%)					
Others (rapidly destructive coxopathy…)	1 (0.52%)	0 (0.00%)	1 (1.45%)		6 (7.06%)	3 (7.50%)	3 (6.67%)	

Data are presented as mean (SD) for continuous variables and as n (%) for categorical variables. *p*-values were calculated using Student’s *t*-test or Mann–Whitney U test for continuous variables and χ^2^ test or Fisher’s exact test for categorical variables, as appropriate. ASA: American Society of Anesthesiologists physical status classification; BMI: Body Mass Index.

## Data Availability

The data presented in this study are available on request from the corresponding author due to ethical and data privacy reasons.

## Supplementary Materials

The following supporting information can be downloaded at: https://www.mdpi.com/article/10.3390/jcm15103585/s1, Ru-SATED & Pittsburgh Sleep Quality Index (PSQI) questionnaire versions used in the study. Table S1. Non-parametric comparisons of PSQI component scores between time points, confirming the robustness of parametric results presented in the main text. Figure S1. Sensitivity analysis excluding unicompartmental knee arthroplasty (UKA): RU-SATED scores by sex for knee arthroplasty patients (TKA only, n = 199). Estimated marginal means (95% CI) from a linear mixed-effects model including visit, sex, and their interaction, with a random intercept for participant. Figure S2. Sensitivity analysis excluding unicompartmental knee arthroplasty (UKA): PSQI scores by sex for knee arthroplasty patients (TKA only, n = 199). Estimated marginal means (95% CI) from a linear mixed-effects model including visit, sex, and their interaction, with a random intercept for participant.

## Abbreviations

The following abbreviations are used in this manuscript:

ASA	American Society of Anesthesiologists
BMI	Body Mass Index
ERAS	Enhanced Recovery After Surgery
KA	Knee Arthroplasty
M_−2_	Two months preoperatively
M_0_	Time of surgery and hospitalisation
M_1_	One month postoperatively
M_6_	Six months postoperatively
MCID	Minimally Clinically Important Difference
OSA	Obstructive Sleep Apnoea
PSQI	Pittsburgh Sleep Quality Index
RU-SATED	Regularity, Satisfaction, Alertness, Timing, Efficiency, Duration (sleep health scale)
SD	Standard Deviation
THA	Total Hip Arthroplasty
TJA	Total Joint Arthroplasty
TKA	Total Knee Arthroplasty
UKA	Unicompartmental Knee Arthroplasty
