# Supplementary material for: Sleep Quality and Sleep Health Before and After Hip or Knee Arthroplasty: A Prospective Cohort Study
Source: J Clin Med. 2026 May 7;15(10):3585. doi: 10.3390/jcm15103585 (PMC13207377; doi:10.3390/jcm15103585)
Supplement: Supplementary file 1 [file jcm-15-03585-s001.zip › jcm-4241029-supplementary.pdf]

ONLINE SUPPLEMENT

Sleep Quality and Sleep Health Before and After Hip or Knee Arthroplasty: A Prospective Cohort Study

Jordi Colomina Morales, Adriano D. S. Targa, Mario Henríquez-Beltrán, Esther Gracia-Lavedán, Iván Juez-Garcia and Jordi de Batlle

Contents

1. Ru-SATED & Pittsburgh Sleep Quality Index (PSQI) versions used in the study 2

    Ru-SATED 2

    PSQI 3

2. Sensitivity analysis: Wilcoxon signed-rank tests for PSQI components 5

3. Sensitivity analysis excluding unicompartmental knee arthroplasty (UKA) 6

    Ru-SATED 6

    PSQI 7

1. Ru-SATED & Pittsburgh Sleep Quality Index (PSQI) versions used in the study

Ru-SATED

¿Con qué frecuencia ha experimentado o realizado cada una de estas situaciones durante la última semana?

1. ¿Está satisfecho/a con su sueño?

Nunca	Casi nunca	A veces	Bastante frecuente	Siempre
☐	☐	☐	☐	☐

2. ¿Permanece despierto/a todo el día sin quedarse dormido/a? (no incluye la siesta)

Nunca	Casi nunca	A veces	Bastante frecuente	Siempre
☐	☐	☐	☐	☐

3. ¿Duerme (o intenta dormir) entre las 2 y las 4 de la madrugada?

Nunca	Casi nunca	A veces	Bastante frecuente	Siempre
☐	☐	☐	☐	☐

4. Por la noche, ¿pasa menos de 30 minutos despierto? (incluye el tiempo que pasa para quedarse dormido y los despertares nocturnos)

Nunca	Casi nunca	A veces	Bastante frecuente	Siempre
☐	☐	☐	☐	☐

5. ¿Duerme entre 6 y 8 horas al día? (incluyendo la siesta)

Nunca	Casi nunca	A veces	Bastante frecuente	Siempre
☐	☐	☐	☐	☐

6. ¿Se acuesta y se levanta a la misma hora todos los días? (con menos de una hora de diferencia)

Nunca	Casi nunca	A veces	Bastante frecuente	Siempre
☐	☐	☐	☐	☐

Reference: Benítez I, Roure N, Pinilla L, Sapiña-Beltran E, Buysse DJ, Barbé F, de Batlle J. Validation of the Satisfaction, Alertness, Timing, Efficiency and Duration (SATED) Questionnaire for Sleep Health Measurement. Ann Am Thorac Soc. 2020 Mar;17(3):338-343. doi: 10.1513/AnnalsATS.201908-628OC.

PSQI

Las siguientes preguntas hacen referencia a cómo ha dormido usted normalmente DURANTE EL ÚLTIMO MES. Intente ajustarse en sus respuestas de la manera más exacta posible a lo ocurrido durante la mayor parte de los días y noches del último mes.

1. **Durante el último mes ¿cuál ha sido, normalmente, su hora de acostarse? __________**
2. **¿Cuánto tiempo habrá tardado en dormirse, normalmente, las noches del último mes? __________**
3. **Durante el último mes, ¿a qué hora se ha levantado habitualmente por la mañana? __________**
4. **¿Cuántas horas calcula que habrá dormido verdaderamente cada noche durante el último mes? __________**

**5. Durante el último mes, cuántas veces ha tenido problemas para dormirse a causa de:**	**Ninguna vez en el último mes**	**Menos de una vez a la semana**	**Una o dos veces a la semana**	**Tres o más veces a la semana**
No poder conciliar el sueño en la primera media hora				
Despertarse durante la noche o de madrugada				
Tener que levantarse para ir al servicio				
No poder respirar bien				
Toser o roncar ruidosamente				
Sentir frío				
Sentir demasiado calor				
Tener pesadillas o malos sueños				
Sufrir dolores				
Otras opciones				

6. **Durante el último mes, ¿cuántas veces habrá tomado medicinas (por su cuenta o recetadas por el médico) para dormir?**
   a. Ninguna vez en el último mes
   b. Menos de una vez a la semana
   c. Una o dos veces a la semana
   d. Tres o más veces a la semana

7. **Durante el último mes, ¿cuántas veces ha sentido somnolencia mientras conducía, comía o desarrollaba alguna otra actividad?**
    a. Ninguna vez en el último mes
    b. Menos de una vez a la semana
    c. Una o dos veces a la semana
    d. Tres o más veces a la semana

8. **Durante el último mes, ¿ha representado para usted mucho problema el tener ánimos para realizar alguna de las actividades detalladas en la pregunta anterior?**
    a. Ningún problema
    b. Un leve problema
    c. Un problema
    d. Un grave problema

9. **Durante el último mes, ¿cómo valoraría en conjunto, la calidad de su sueño?**
    a. Muy buena
    b. Bastante buena
    c. Bastante mala
    d. Muy mala

10. **¿Duerme usted solo o acompañado?**
    a. Solo
    b. Con alguien en otra habitación
    c. Con alguien en la misma habitación, pero en otra cama
    d. Con alguien en la misma cama

Reference: Macías Fernández JA, Royuela Rico A. La versión española del índice de calidad de sueño de Pittsburgh. Informaciones Psiquiátricas 1996; 146: 465-472.

2. Sensitivity analysis: Wilcoxon signed-rank tests for PSQI components

Table S1. Non-parametric comparisons of PSQI component scores between time points, confirming the robustness of parametric results presented in the main text.

PSQI Component	M_{-2} Median (Q1, Q3) N = 259	M_1 Median (Q1, Q3) N = 236	M_6 Median (Q1, Q3) N = 204	M_{-2} vs M_1 p-value n = 236	M_{-2} vs M_6 p-value n = 204	M_1 vs M_6 p-value n = 181
Subjective sleep quality	1.00 (1.00, 1.00)	1.00 (1.00, 2.00)	1.00 (0.00, 1.00)	>0.9	<0.001	<0.001
Sleep latency	1.00 (0.00, 2.00)	1.00 (0.00, 2.00)	1.00 (0.00, 1.00)	<0.001	0.3	<0.001
Sleep duration	0.00 (0.00, 1.00)	0.00 (0.00, 1.00)	0.00 (0.00, 0.00)	0.13	0.008	<0.001
Habitual sleep efficiency	0.00 (0.00, 1.00)	0.00 (0.00, 2.00)	0.00 (0.00, 1.00)	0.14	<0.001	<0.001
Sleep disturbances	1.00 (1.00, 1.00)	1.00 (1.00, 1.00)	1.00 (1.00, 1.00)	0.040	0.014	0.9
Use of sleep medication	0.00 (0.00, 0.00)	0.00 (0.00, 1.00)	0.00 (0.00, 0.00)	>0.9	0.020	0.007
Daytime dysfunction	0.00 (0.00, 1.00)	0.00 (0.00, 0.00)	0.00 (0.00, 0.00)	0.2	0.065	0.2

All p-values from Wilcoxon signed-rank test with continuity correction. Conclusions are consistent with parametric analyses across all components.

3. Sensitivity analysis excluding unicompartmental knee arthroplasty (UKA)

Ru-SATED

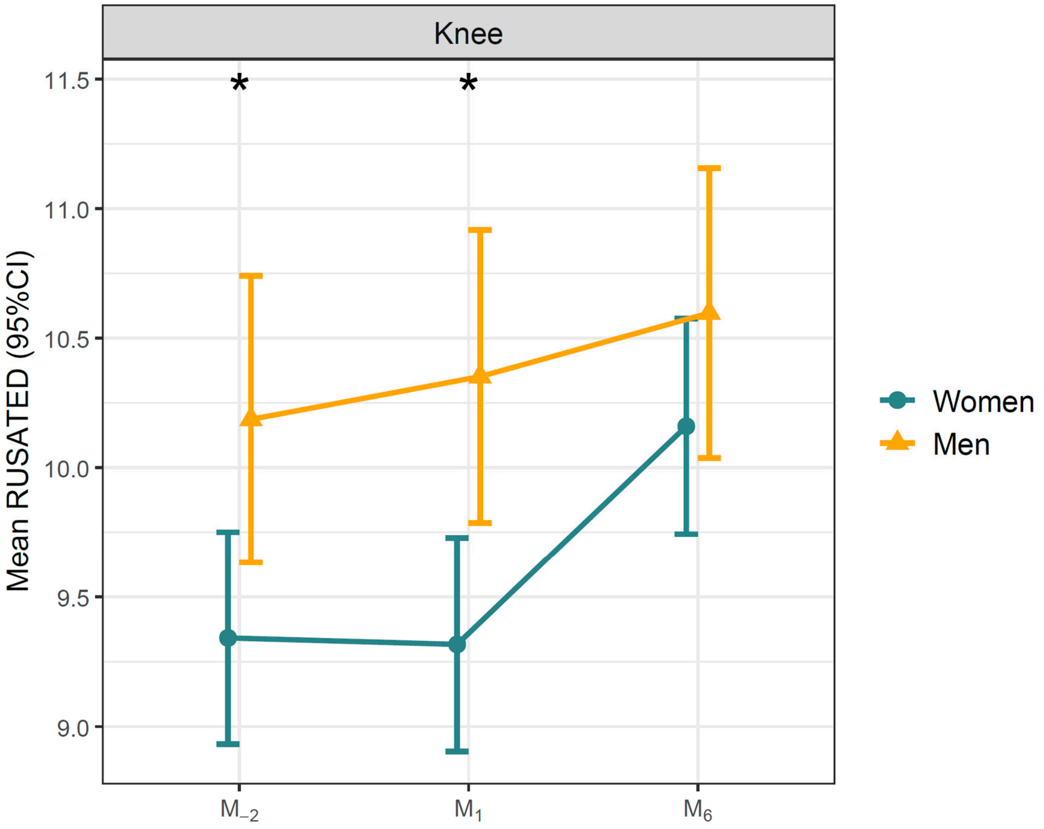

Figure S1. Sensitivity analysis excluding unicompartmental knee arthroplasty (UKA): RU-SATED scores by sex for knee arthroplasty patients (TKA only, n = 199). Estimated marginal means (95% CI) from a linear mixed-effects model including visit, sex, and their interaction, with a random intercept for participant.

PSQI

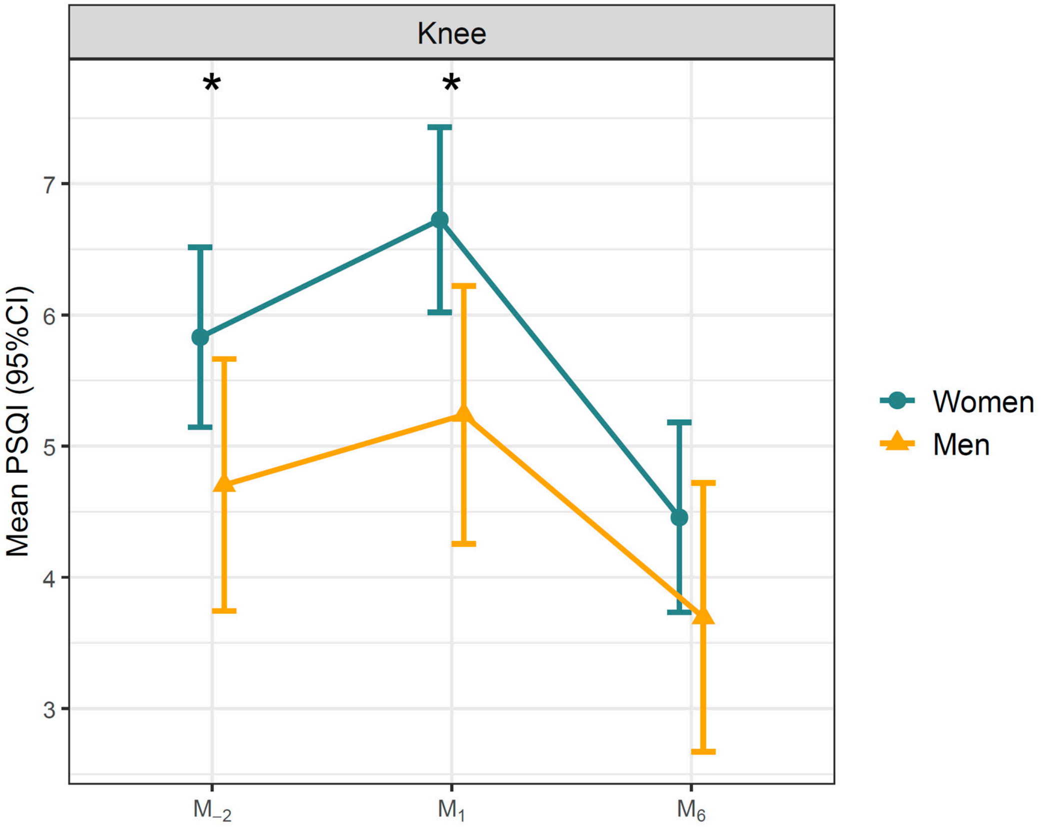

Figure S2. Sensitivity analysis excluding unicompartmental knee arthroplasty (UKA): PSQI scores by sex for knee arthroplasty patients (TKA only, n = 199). Estimated marginal means (95% CI) from a linear mixed-effects model including visit, sex, and their interaction, with a random intercept for participant.
